# Responsibilities for receiving and using individual participant data

**DOI:** 10.1002/cesm.12028

**Published:** 2023-11-03

**Authors:** Kylie E. Hunter, Aidan C. Tan, Angela C. Webster, Daniel G. Hamilton, Adrian Barnett, Lee Jones, Myra Cheng, Salma Fahridin, Antonio Laguna‐Camacho, Sol Libesman, Mark Hoffmann, Rui Wang, Anna Lene Seidler

**Affiliations:** ^1^ NHMRC Clinical Trials Centre University of Sydney Sydney New South Wales Australia; ^2^ MetaMelb Research Group University of Melbourne Melbourne Victoria Australia; ^3^ Melbourne Medical School University of Melbourne Melbourne Victoria Australia; ^4^ School of Public Health and Social Work Queensland University of Technology Brisbane Queensland Australia; ^5^ Australian Research Data Commons Canberra Australian Capital Territory Australia; ^6^ School of Medicine Autonomous University of the State of Mexico Toluca Mexico; ^7^ Queensland Cyber Infrastructure Foundation Brisbane Queensland Australia; ^8^ Department of Obstetrics and Gynaecology Monash University Melbourne Victoria Australia

**Keywords:** data provider, data recipient, data sharing, health research, individual participant data, reproducibility

## Abstract

**Background:**

Sharing of individual participant data enhances the value of existing data to generate new evidence and inform decision‐making. While there is strong in‐principle support for data sharing, in practice study data are often difficult to find, access, and re‐use. Currently, there is no consensus statement to guide the data‐sharing process. In particular, more guidance is needed on the responsibilities of data recipients for re‐using individual participant data.

**Purpose:**

To determine views on the responsibilities of recipients of study data, and to propose how these responsibilities could be met.

**Methods:**

A 2‐h online focus group was conducted at the 2021 Association for Interdisciplinary Meta‐research and Open Science conference. Three example data‐sharing scenarios were discussed (evidence synthesis, study reproducibility, and secondary analyses). Notes and audio transcripts were collated using thematic analysis and shared with attendees for further iterative input.

**Results:**

A purposive sample of 16 conference delegates attended the focus group. Analyses revealed four recurring themes that were synthesized into recommendations. The “privacy and ethics” theme described the need for data recipients to prioritize the protection of participant privacy, and the recommendation to proactively share a secure data management plan and evidence of ethical oversight with the data provider. The “capability and resourcing” theme required recipients to demonstrate sufficient capacity to process and analyze study data. The “recognition and collaboration” theme asserted the responsibility to acknowledge the contributions of data providers and invite them to contribute to the secondary project. Last, the “compliance” theme focused on the responsibility to adhere to local data sharing regulations.

**Conclusions:**

Successful data sharing and re‐use requires cooperation from multiple stakeholders. We identified the responsibilities of recipients of study data to the individual from whom data arose and the research team who collected the data. Implementation of these in practice could facilitate increased data sharing.

## 1 INTRODUCTION

**Figure 1:**
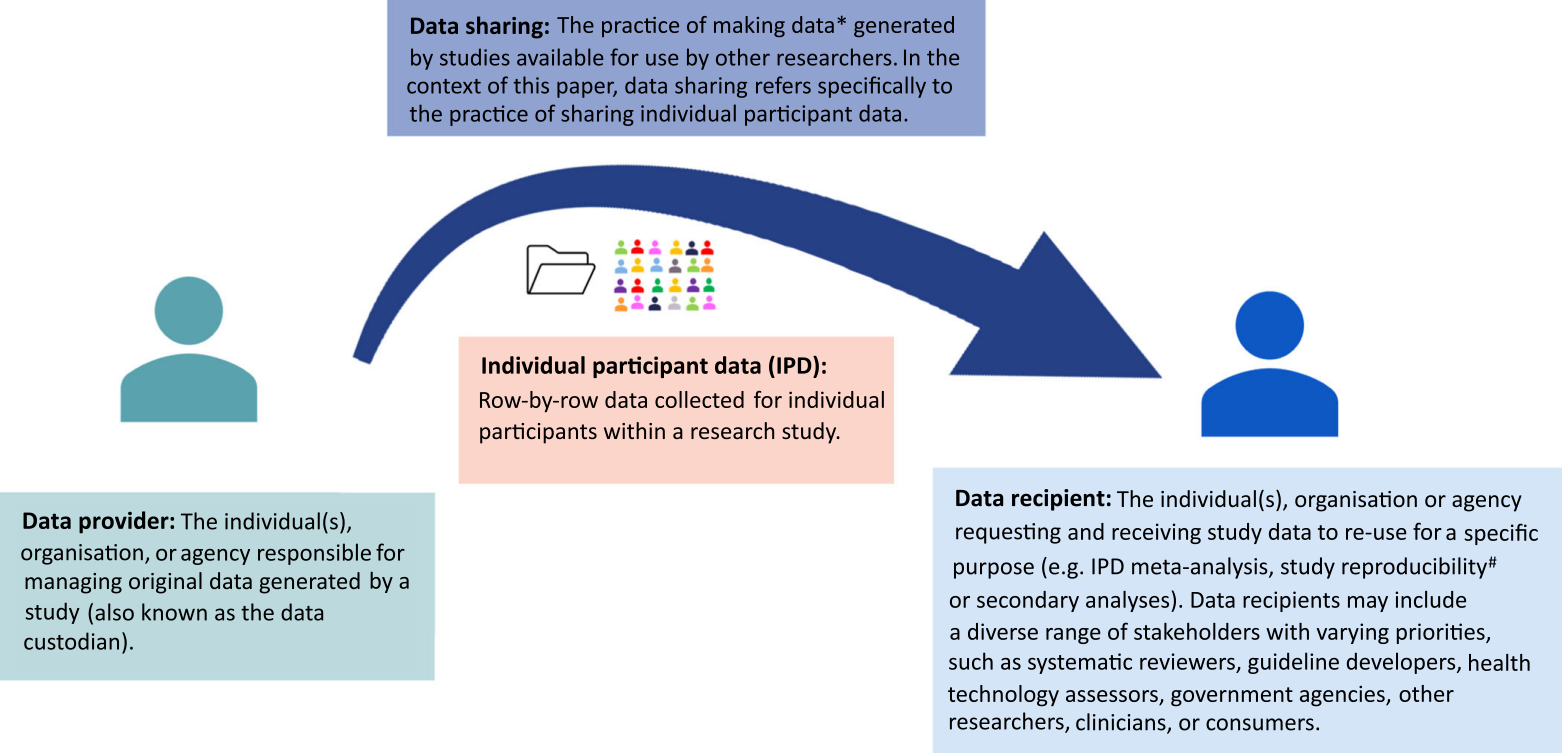
Data sharing process. *(Scientific) data: characteristics or information, usually numerical or categorical, that are collected through observation. Herein, we specifically refer to data collected on or from human participants (excluding animal data, geosciences, etc.). ^#^ *Reproducibility*: Whether the reported findings are repeatable using the same analysis on the same data as the original study.

Data sharing is the practice of making data available for use by other researchers. While this may take varying forms, this research paper focuses specifically on sharing individual participant data (IPD) from studies. That is, row‐by‐row, typically deidentified data collected for individual human participants within a health research study. In health research, study data may be shared and re‐used for various purposes, including new research questions, evidence synthesis, reproducibility, and secondary analyses. For instance, researchers can collect and combine study data across similar studies in an IPD meta‐analysis to improve robustness of results and enhance statistical power to detect benefits or harms of interventions [1]. Importantly, access to IPD enables examination of subgroup effects, such as priority populations, and therefore may be a powerful tool to increase equity in research. In reproducibility studies, researchers attempt to verify a study's findings using the same analysis on the original study data [2]. This may strengthen validity of the results, or uncover concerns that need to be addressed [3], as with the reanalysis of GlaxoSmithKline's study on paroxetine and imipramine for the treatment of depression [4]. Conducting secondary analyses on existing datasets to evaluate new important questions increases the utility of the original data for little additional cost [5]. In summary, data re‐use can add tremendous value to previously funded projects by allowing generation of additional knowledge from existing data and by enhancing the credibility of existing findings (Figure 1).

Recently, particularly since the onset of the COVID‐19 pandemic, calls for data sharing have become increasingly prominent. This is in recognition of the benefits of data sharing, as well as an increasing awareness of the extent of research waste and the threats this poses to evidence‐based medicine. For instance, in 2017 the International Committee of Medical Journal Editors declared data sharing an ethical obligation [6], to honor the risk participants take by increasing the likelihood their participation results in useful findings. Furthermore, an increasing number of research funders and journal editors have begun requiring data sharing for studies they fund and publish [7, 8, 9].

However, despite these initiatives and strong in‐principle support for the concept of data sharing in health research, in practice it is often difficult to operationalize [10]. In a recent cross‐sectional study [11] of 487 clinical trials published in *JAMA*, *Lancet*, and *New England Journal of Medicine*, 334 articles (67%) declared an intention to share IPD, but only two (0.6%) had IPD datasets available on a journal website and 17 (5%) on data repositories. A further 143 (43%) declared study data would be accessible via request to authors, yet the success rate for this method is suboptimal [10]. One systematic review found that only 58% of 2745 datasets requested by IPD meta‐analysts were actually retrieved [12], while another reported that only 25% of 760 IPD meta‐analyses retrieved all of the eligible IPD for analysis [13]. Moreover, data retrieval via authors is often a lengthy and resource‐intensive process. A recent case study found the median time from requesting to receiving data was 88 days (IQR = 130) and required an average of 23 emails (IQR = 25) [14]. Others have reported taking up to 4 years to retrieve IPD [15].

To understand this reluctance and delay in sharing data, Tan et al [16]. surveyed researchers and analyzed data sharing plans in trial registration records. They found that barriers to data sharing were multifactorial, including concerns about protecting privacy of participants, data security, inappropriate analyses, data dredging, and uncertainty about how to best undertake data sharing in practice [16]. These concerns have led some to label those who re‐use study data as “research parasites” [17]. Additionally, some trialists thought data sharing could not be resourced from trial funding, and was not integral to trial design, but rather an additional undertaking upon trial completion [16]. Some of these concerns could be alleviated by establishing key responsibilities of data recipients for appropriate re‐use of study data. While there are many data sharing policies and standards across different stakeholders [9], there are no agreed global standards, no guidance for implementation, and little guidance about the role of data recipients.

### 1.1 Purpose

This qualitative study aimed to determine health research stakeholders' views on the responsibilities of those who request and re‐use IPD from studies (i.e., data recipients) in health research. Specifically, the objectives were to determine the focus group's perceptions of duties or obligations that data recipients have toward: (i) the participants who contributed to the research by providing their consent and data; (ii) the researcher(s) who collected, collated, and published the data; and (iii) the data providers who are entrusted to manage the data.

## 2 MATERIALS AND METHODS

### 2.1 Study design

We conducted a 2‐h focus group online on December 3, 2021 as part of the Association for Interdisciplinary Meta‐research and Open Science (AIMOS) conference. The facilitators (Kylie E. Hunter, Anna Lene Seidler, Angela C. Webster, and Aidan C. Tan) specialize in conducting IPD meta‐analyses, and therefore frequently request and receive study data for re‐use and are in favor of data sharing. All had some level of training or experience in qualitative research methods. Three of the facilitators were female and one was male.

### 2.2 Sampling strategy

The AIMOS 2021 conference was open for all to attend and offered free registration, if needed. The focus group was open to all conference delegates, and we used a purposive sampling strategy by sending targeted email invitations to known data sharing experts.

### 2.3 Data collection

The focus group involved a discussion of three case studies developed by the facilitators, each exploring a common IPD sharing scenario (Table 1, Supporting Information: Appendix 1).

**Table 1:** Description of the three case studies discussed at focus group.

Case study 1. Evidence synthesis and protecting study participants	*Researcher A requests IPD from Researcher Z for an IPD meta‐analysis. Researcher Z is concerned about protecting study participants' identities*.
Case study 2. Study reproducibility and ensuring appropriate data use	*Researcher B requests data from Researcher Z for a reproducibility study. Researcher Z is concerned about ensuring appropriate data use*.
Case study 3. Secondary analyses and protecting the interests of data providers	*Researcher C requests data from Researcher Z for secondary analyses. Researcher Z is concerned about protecting their and their funders' interests*.

For each case study, participants were asked to consider:

- what they thought were the responsibilities of data recipients across the design, conduct, analyses, reporting and data re‐use stages of their research;
- current practices and principles they follow when requesting and re‐using data;
- barriers faced; and,
- how they could address raised barriers and promote responsible re‐use of data.

Participants contributed by speaking in the video call or by typing real‐time on shared Google Documents. There was no one else present beside the participants and facilitators. Basic demographic information, including field of work/research, career stage, primary employer/affiliation, and data request history were collected via Slido surveys.

### 2.4 Data processing and analysis

Video and audio of the discussion were recorded with the participants' consent, and transcribed verbatim. Transcripts and textual data from group notes were analyzed using an inductive thematic approach [18, 19]. After familiarization with these data, the first authors (Kylie E. Hunter and Aidan C. Tan) generated initial codes in an electronic word‐processing file, focusing on a descriptive and semantic level of meaning appropriate to the research question. Next, codes were mapped into distinctive and coherent patterns and used to construct themes. Findings and transcripts were shared with participants, who were invited to review and refine the themes. All comments and responses were anonymised. This study has been reported in accordance with the Standards for Reporting Qualitative Research (Supporting Information: Appendix 2) [20] and Consolidated Criteria for Reporting Qualitative Studies (COREQ) [21].

## 3 RESULTS

Sixteen conference delegates participated in the focus group, including four facilitators (Supporting Information: Appendix 3 shows participant demographics), with no refusals or drop‐outs. The majority of participants (*n* = 11, 69%) cited a university as their primary employer and 15 were based in Australia and one in Mexico. AIMOS conferences are multidisciplinary and while all participants had some link to health research, they worked across a broad range of fields, including epidemiology, statistics, evidence synthesis, policy, ethics, meta‐research, public health, data management, oncology, psychology, nutrition, indigenous health, clinical trials, and administration. Participants included early (*n* = 6, 38%), mid (*n* = 2, 13%) and senior (*n* = 2, 13%) career researchers, a data policy officer (*n* = 1, 6%) and a senior health informatician (*n* = 1, 6%). Four (25%) did not report their role. Half the participants had previously requested data from another investigator for re‐use. Reasons varied and included IPD meta‐analyses, aggregate data meta‐analysis, prospective meta‐analyses, network meta‐analyses, secondary analyses, “many analysts”‐style research (i.e., checking the robustness of results when using other analysis strategies), simulation‐based sample size calculations, power analyses, replication, reproducibility checks, and other purposes.

### 3.1 Synthesis

**Figure 2:**
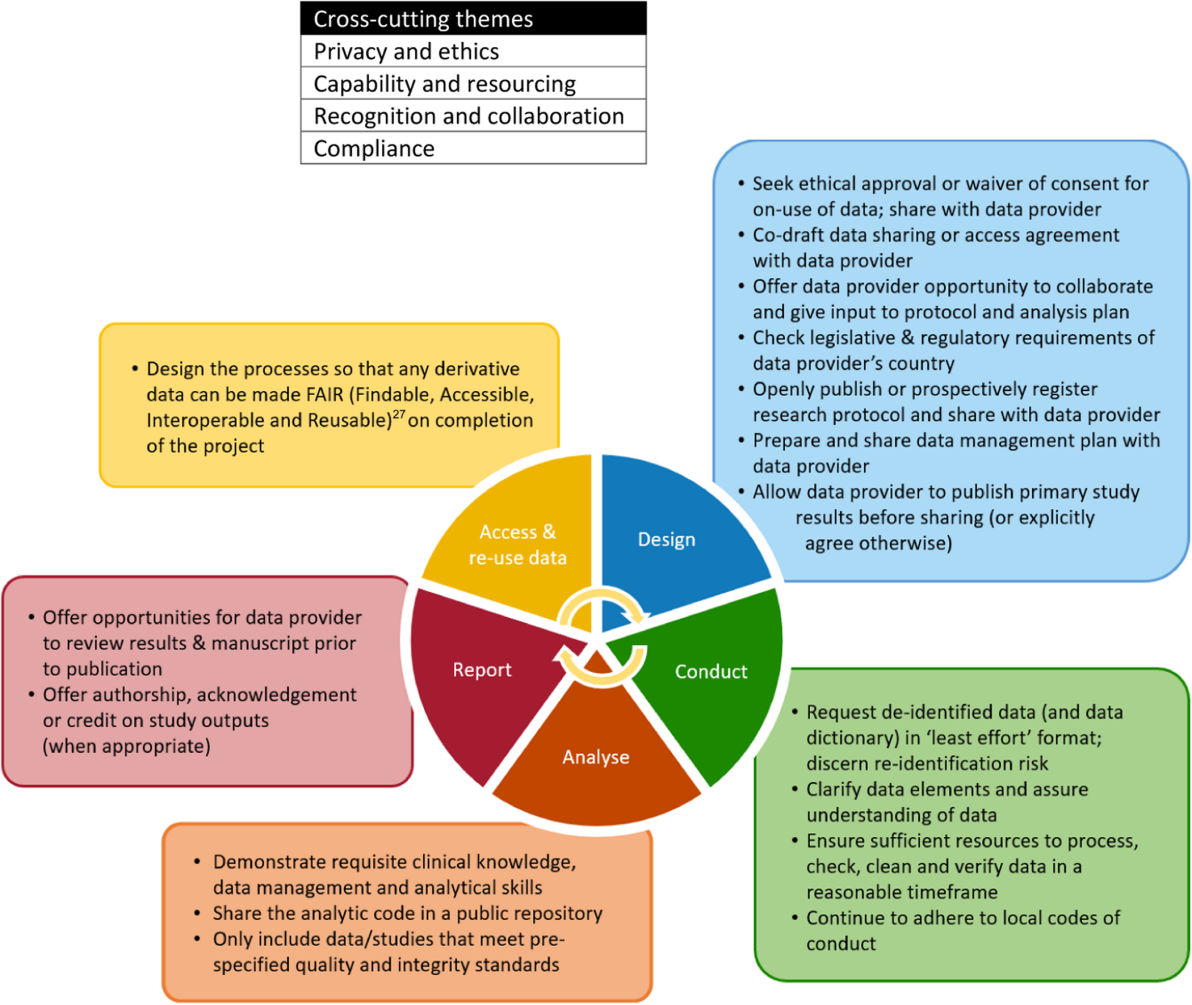
Responsibilities of data recipients for secondary use of study data across each stage of the health research cycle.

**Figure 3:**
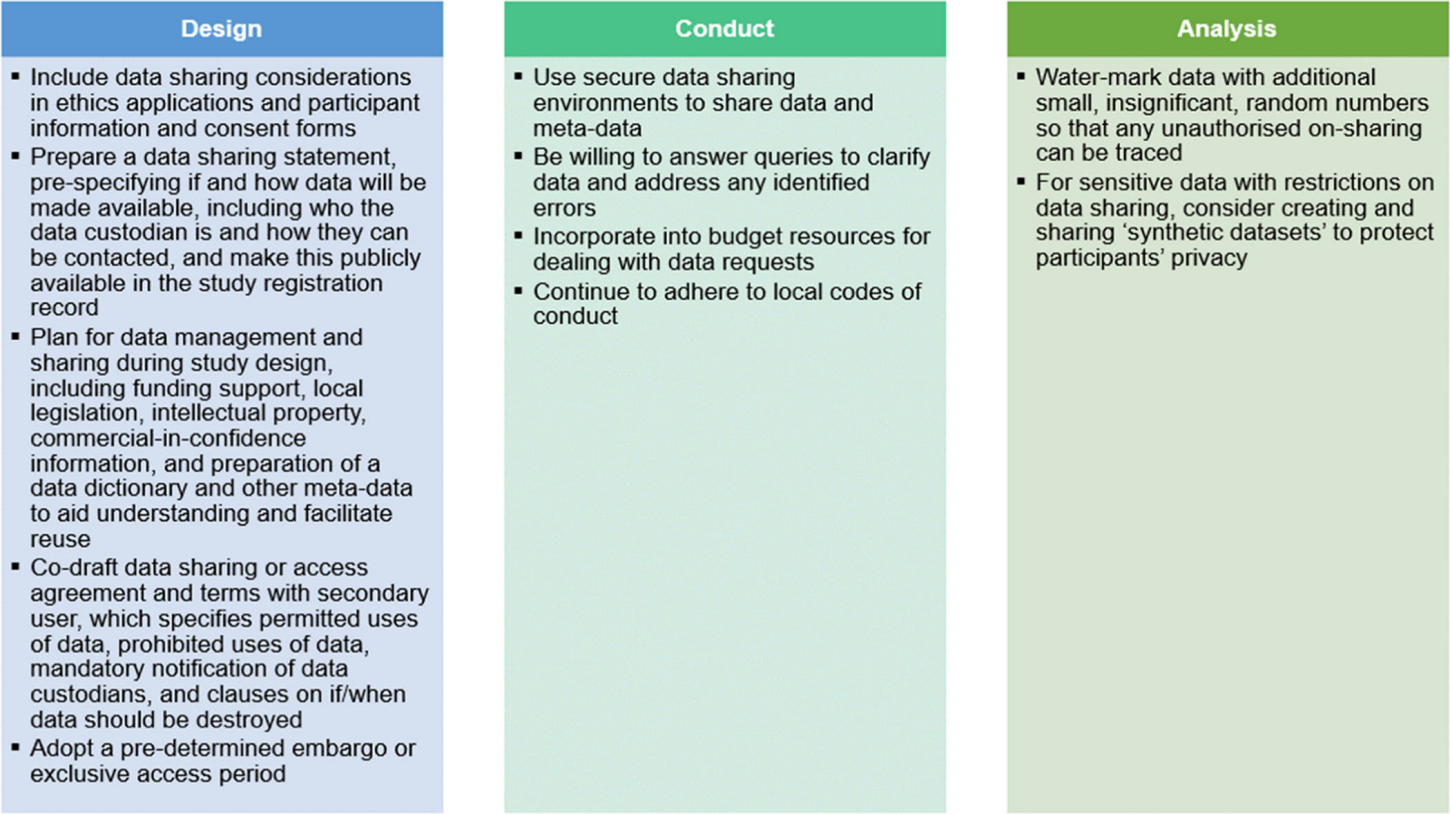
How data providers can support data recipients to meet their responsibilities by stage of study.

Four key themes emerged from discussion for the responsibilities of data recipients across the design, conduct, analysis, reporting and re‐use stages of health research (Figure 2). Most of these are early in the research design stage, demonstrating the need for data recipients to prospectively plan how to meet their responsibilities. Although the focus of discussion was on the responsibilities of data recipients, participants also identified themes for ways in which data providers could support data recipients to meet their responsibilities, and we report on these as well (Figure 3). A key cross‐cutting theme was the importance of recognition of the data provider (e.g., data authorship) and a collaborative approach between data recipient and provider throughout all stages of the research cycle. Other key themes included privacy and ethics, capability and resourcing, and compliance with data sharing rules or regulations. Each of these are elaborated below, with accompanying recommendations for data recipients. The group recognized that applicability of items may vary depending on the level of access to study data, such as whether study data are publicly available to everyone or via a moderated access policy (i.e., the data provider arbitrates data availability).

## 4 THEME: PRIVACY AND ETHICS

### 4.1 Responsibility: Ensure adequate ethics approval and informed consent

Concerns about ethics approval and informed consent are commonly cited barriers to data sharing [16]. Data providers are often hesitant to share study data without specific provision for this in their ethics approval, or when participants have not explicitly consented for their study data to be used by others. We determined that a key responsibility of data recipients to alleviate these concerns is to seek ethics approval or a waiver of consent to receive and re‐use study data, and provide a copy of this to the data provider. Data providers could support this by including data sharing provisions in ethics applications and participant information consent forms as standard practice.

“Were subsequent studies or data sharing included in the Trial participant agreement/consent form?” “What did your original consent form say about how the data would be used?”

### 4.2 Responsibility: Protect participant privacy and confidentiality

Additional major barriers to data sharing were concerns about protecting participant privacy and confidentiality [16, 22]. Focus group participants expressed concern that receiving and re‐using IPD from studies could result in participant identification and risk their privacy and confidentiality. This included specific concerns about sensitive and small study populations. It was noted that participant identification or major public health harm from shared data is very rare, and that the secondary use of study data which received ethics approval for its primary use was unlikely to introduce new ethical concerns.

We identified several responsibilities for data recipients to address these concerns. First, drafting data sharing or access agreements with terms of use which specify permitted and prohibited uses of study data, include mandatory notification of data breaches to data providers, clauses about on‐sharing and destruction of study data, and roles and responsibilities of data providers and data recipients (e.g., data security). This could include a written declaration that recipients will not attempt to reidentify the study data or participants (such as already in use by data repositories [23]). Data providers may also wish to uniquely watermark their study data for each recipient by adding small, random numbers (e.g., a single random digit to the third decimal place) for a subset of values so there is no effect on any analyses. This would create an accountability trail leading back to the data recipient if the study data are shared by the data recipient with other investigators.

Second, data recipients should only request deidentified data [24] (i.e., study data without any personal identifiers). To aid this process, data recipients could provide specific instructions to data providers on the likely types of variables with participant identifiers and re‐identifiable information. They could inform on the likely risks of participant re‐identification, especially if linked to other datasets. Third, data recipients should share their data management plan and statistical analysis plan with the data provider to demonstrate adherence to secure procedures and capability to correctly analyze the study data. Other options to adhere to this responsibility are to conduct all analyses on the data provider's site, or on a trusted research environment (TRE), also known as a secure data environment. TREs provide secure access for approved researchers to conduct statistical analyses remotely, so that raw data never leave the environment [25].

I think if I was owning the data and somebody asked to share it with me, I'd want to see their statistical analysis plan, their protocol, their ethics approval, and understand clearly what their data storage and access and use arrangements were.

In cases where data sharing is refused, a proposed solution was for data providers to run the statistical analysis with code provided by the data recipient (e.g., calculating the required summary statistics for a systematic review) and providing the results to the data recipient (e.g., OpenSAFELY (https://docs.opensafely.org/) for NHS electronic health records). In these instances, data providers could support data recipients by providing them with a scrambled (but realistic) data set to develop their code. Alternatively, data providers may consider creating a synthetic data set, which mimics the real data set by maintaining the statistical properties and relationships between variables, but avoids disclosing the original values [26]. Since the data do not represent any real individuals, participant privacy and confidentiality are safeguarded. However, these solutions frequently limit the analyses and checks that can be undertaken with the data, and place considerable burden on the data provider. Therefore, they are often not feasible nor desired.

## 5 THEME: CAPABILITY AND RESOURCING

### 5.1 Responsibility: Avoid misinterpretation of the data and misleading secondary analyses

Researchers are often concerned that data recipients will incorrectly interpret their study data or, intentionally or unintentionally, conduct inappropriate or misleading secondary analyses. Communication, collaboration and transparency are key to addressing these concerns. Communication with data providers allows data recipients to demonstrate they have the requisite clinical knowledge, data management and analytic skills to appropriately re‐use study data, and ensure they understand the study data they receive. For instance, data recipients could request the data dictionary and analytic code from the original study and clarify any unclear data elements with the data provider. Conversely, communication with data recipients would allow data providers (within reason) to assist with data queries and aid understanding of the data set. For the full benefits of shared data to be realized, data providers should make their data FAIR (findable, accessible, interoperable and re‐usable^1^ [27]).

Collaboration could be initiated by data recipients offering data providers an opportunity to provide early input on the conceptual framework of their study at the outset, and to review the protocol and statistical analysis plan. Transparency could be achieved by openly publishing the research protocol in a peer‐reviewed journal or prospectively registering it in a public registry, and by sharing the statistical analysis plan and analytic code in a public repository. Later, data providers may be given the opportunity to provide feedback on the interpretation of results and content of manuscripts.

… what's really Important is understanding the origin of the data. So having the original data custodians to be able to ask them questions and collaborate with them is a good idea.

### 5.2 Responsibility: Allow adequate resourcing to support data sharing

Preparing original study data for sharing may be onerous for data providers, particularly for older studies managed by staff who have retired or moved on, or those with limited documentation. Data recipients have a responsibility to ease this burden on data providers by requesting study data in the ‘least effort’ format, and then converting them to the most useable format.

My perspective is it is an obligation of trialists to ensure their data continues to be used and it should be designed in… If you design it properly, then it's not that hard.

## 6 THEME: RECOGNITION AND COLLABORATION

### 6.1 Responsibility: Ensure sufficient academic/scientific recognition for data sharing

Data providers may be hesitant to share their data, particularly if they have not yet published the results, because they fear their contributions will not receive appropriate recognition. Data recipients have a responsibility to ensure the data provider receives appropriate recognition. This could be done by offering authorship (on condition of input to the final paper), acknowledgment or credit on the study publication or other outputs, and citing publicly available datasets, in accordance with relevant guidelines (e.g., ICMJE guidelines) [28]. Additionally, data recipients should allow data providers appropriate opportunity to publish their own results first, because some journals value novelty and may be hesitant to accept something they view as a secondary publication. To achieve this, they could negotiate an agreement with data providers to withhold publication of the secondary study until a predetermined embargo period has elapsed (e.g., after publication of primary analysis).

More broadly, participants argued that the academic system needs to be re‐structured to value data sharing and secondary analyses as important criteria for grants and promotions. Some thought that shared data or data articles (scientific publications that describe datasets) [29] should be acknowledged as research outputs that can be readily cited, a concept known as “data authorship.” Promoting how data providers may benefit from data sharing via increased citations, and recognition of the original study may facilitate increased willingness to share data.

… there has to be a benefit for everyone involved … so if the decision is made that there is benefit to the people who own the data or who hold the data that they will share it.

“I think if there was proper incentives and proper funding, this would all be a lot easier.”

### 6.2 Responsibility: Protect commercially sensitive information and funder agreements

Participants identified concerns that data sharing may breach formal agreements with sponsors, funders or collaborators. This may adversely affect commercial‐in‐confidence information, product development, intellectual property, patent filings, or regulatory approval, licensure, or clearance. To mitigate this concern, data recipients should seek formal approval from the relevant sponsors, funders, or collaborators to re‐use their study data. A potentially complicating factor is defining the data custodian, especially for industry studies, since multiple stakeholders are often involved (e.g., researchers, data analysts, clinical/industry experts, government agencies). Each may be subject to varying laws and regulations around data ownership.

Other factors: … Intellectual Property concerns for any commercially funded trials…; Check the licensing terms of publicly available data

## 7 THEME: COMPLIANCE

### 7.1 Responsibility: Comply with data sharing regulations across locations

Since data sharing is an emerging practice, the rules and regulations governing it are evolving. The ease or difficulty of obtaining study data can be influenced by who collected the study data, the location and nationality of participants, and the location of the data recipient. Participants agreed it should be the joint responsibility of data providers and data recipients to familiarize themselves and comply with local data privacy and protection legislation, as well as relevant codes of conduct (e.g., from universities or professional societies).

[Recipients] should be bound by their codes of conduct, e.g., those from their university and professional society.

## 8 DISCUSSION

Our study brought together experts with diverse data sharing experiences. Participants identified a wide range of barriers and concerns regarding data sharing from the perspective of both the data recipient and data provider. Each issue was discussed in the context of different case studies, and practical solutions and strategies to address them were derived by robust discussion.

In particular, we derived several responsibilities of data recipients. Recipients should produce and publicize data management plans and involve data providers in this process. These data management plans should describe how data recipients will protect study participants, ensure appropriate data use, and protect the interests of data providers. Data recipients should be able to demonstrate competency and expertise to protect against misinterpretation or misuse of study data. They also need to be aware of restrictions imposed on data providers regarding data sharing (e.g., commercial), and work together to negotiate appropriate solutions.

### 8.1 How can we ensure that data sharing responsibilities are met?

There is an urgent need to operationalize solutions to ensure the identified responsibilities are met. There is a lack of agreed standards for preparing, sharing, accessing, and re‐using research data. A recent study [9] found key stakeholders had limited requirements or guidance to share data, suggesting uncertainty about who is responsible for its enforcement. Implementing standard data sharing policies across key stakeholders, including funders, ethics committees, trial registries, government bodies, journals and data repositories, may facilitate improved data sharing knowledge and behavior change in researchers.

Building appropriate infrastructure is essential to improve data sharing. There are now many commercial and noncommercial data sharing repositories. However, lodgement costs, access processes, and sustainability are neither standardized nor secured. In Australia, the Health Studies Australian National Data Asset (HeSANDA, https://ardc.edu.au/program/health-studies-australian-national-data-asset/) program is addressing this issue by building national infrastructure for sharing and reusing study data.

Another important consideration is who will resource and fund data sharing activities, which are often poorly planned, costly, labor‐intensive, and require specific expertise. There is a need to streamline these activities and incorporate them into research funding as standard practice. For instance, it has been proposed that 5% of grant funds should go toward data stewardship [30]. Specific funding schemes that encourage data re‐use may also provide support, for example, Restoring Invisible and Abandoned Trials Support Grants [31].

### 8.2 Strengths

A key strength and novel aspect of this study was the focus on responsibilities of data recipients, in contrast to the more commonly emphasized responsibilities of data providers. Further, the focus group format enabled interactive discussion, generation of ideas, and in‐depth exploration of the experiences and views of the range of experts involved. Notably, focus group participants had previously requested study data for a diverse range of reasons, and therefore their perspectives may have wide application.

### 8.3 Limitations

Our findings may not be generalizable internationally and may not incorporate the perspectives of all stakeholders. However, we included participants with a range of experience in re‐using study data for different scenarios, from a range of primary employers and roles (including researchers and non‐researchers), across all career stages (early, mid and senior) and a variety of fields. Future research should seek to capture the range of experience within a sampling frame and triangulate multiple sources of study data.

The facilitators are in favor of data sharing, and were thus cognizant of their potential assumptions or presuppositions throughout this study—when designing the research questions, approach and methods, and discussing the results and transferability. Moreover, participants at the AIMOS conference may be sympathetic to data sharing. Future research should seek to capture alternative or contrasting perspectives.

## 9 CONCLUSION

Health research represents a huge investment of public funding, research time, and participant trust. Re‐using data collected in these studies could maximize investment by enabling researchers to answer new research questions, and to address issues of replication and reproducibility. We have identified several responsibilities of data recipients when requesting and reusing IPD from studies. These responsibilities need to be further delineated and operationalized to enable existing strong support for the principle of data sharing to be translated into research practice.

## AUTHOR CONTRIBUTIONS

**Kylie E. Hunter**: Conceptualization; data curation; formal analysis; investigation; methodology; project administration; resources; validation; visualization; writing—original draft; writing—review and editing. **Aidan C. Tan**: Conceptualization; data curation; formal analysis; investigation; methodology; project administration; resources; validation; visualization; writing—original draft; writing—review and editing. **Angela C. Webster**: Conceptualization; investigation; methodology; resources; supervision; writing—review and editing. **Daniel G. Hamilton**: Investigation; resources; validation; writing—review and editing. **Adrian Barnett**: Investigation; resources; validation; writing—review and editing. **Lee Jones**: Investigation; resources; validation; writing—review and editing. **Myra Cheng**: Investigation; resources; validation; writing—review and editing. **Salma Fahridin**: Investigation; resources; validation; writing—review and editing. **Antonio Laguna‐Camacho**: Investigation; resources; validation; writing—review and editing. **Sol Libesman**: Investigation; resources; validation; writing—review and editing. **Mark Hoffmann**: Investigation; resources; validation; writing—review and editing. **Rui Wang**: Investigation; resources; validation; writing—review and editing. **Anna Lene Seidler**: Conceptualization; data curation; formal analysis; investigation; methodology; project administration; resources; supervision; validation; visualization; writing—review and editing.

## CONFLICTS OF INTEREST STATEMENT

Adrian Barnett is the President of the Association for Interdisciplinary Meta‐Science and Open Science (AIMOS), Daniel G. Hamilton is a Board Member of AIMOS, and Aidan C. Tan is the Treasurer and a Board Member of AIMOS. AIMOS is a not‐for‐profit organization, and these roles are unpaid. Myra Cheng is employed by the Australian Research Data Commons (ARDC). The ARDC is a not‐for‐profit company limited by guarantee.

## ETHICS STATEMENT

Ethical oversight was not sought for this conference session with co‐investigators.

## Supporting information

Supporting information.

Supporting information.

Supporting information.

## Data Availability

The deidentified transcript underlying results of this study and the Microsoft PowerPoint presented at the focus group are openly available in Open Science Framework at https://osf.io/2QHVT/, Identifier: doi:10.17605/OSF.IO/2QHVT.

## Footnotes

^1^
We note that following the development of the CARE Principles for Indigenous Data Governance (Collective Benefit, Authority to Control, Responsibility and Ethics) in 2018, Indigenous scholars in the US published the CARE Principle Data Primer (“CARE Data Principles, Indigenous Data, Data related to Indigenous Peoples and Interest”) in 2023: Barsness (2023). CARE Data Principles, Indigenous Data, Data related to Indigenous Peoples and Interest. Data Curation Network. Retrieved from the University of Minnesota Digital Conservancy, https://conservancy.umn.edu/handle/11299/256919. Comparable developments are underway in Australia. For example, in the context of national research infrastructure, the Indigenous Data Network (IDN), in partnership with the Australian Research Data Commons (ARDC) and other stakeholders are delivering an Indigenous research capability program that celebrates, supports and enhances the capabilities of Aboriginal and Torres Strait Islander peoples and researchers at the interface of research data science and Indigenous knowledge systems. For more information on this collaboration, see ARDC, Improving Indigenous Research Capability: An Aboriginal and Torres Strait Islander Data Commons https://ardc.edu.au/project/improving-indigenous-research-capability/.
