# Supplementary material for: Responsibilities for receiving and using individual participant data
Source: Cochrane Evid Synth Methods. 2023 Nov 3;1(9):e12028. doi: 10.1002/cesm.12028 (PMC11795960; doi:10.1002/cesm.12028)
Supplement: Supplementary file 1 — Supporting information. [file CESM-1-e12028-s001.pdf]

Case 1: Evidence synthesis and protecting study participants

I am conducting an individual participant data meta-analysis, and your trial is eligible to contribute. Can you please share your raw trial data for inclusion?

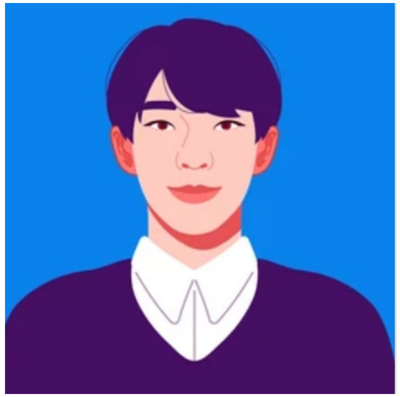

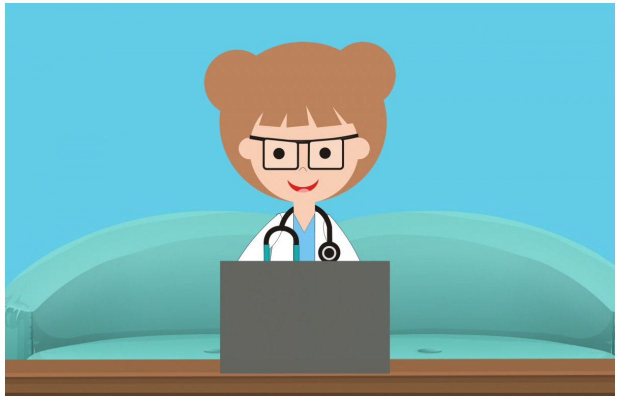

Great idea, I would love to contribute! But I am worried about protecting trial participants. How will you prevent participant identification and protect their privacy and confidentiality?

Case 2: Study replication and ensuring appropriate data use

I am interested in replicating your study. Would you be willing to share your data for this purpose?

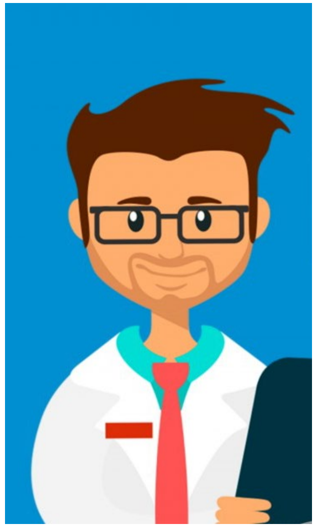

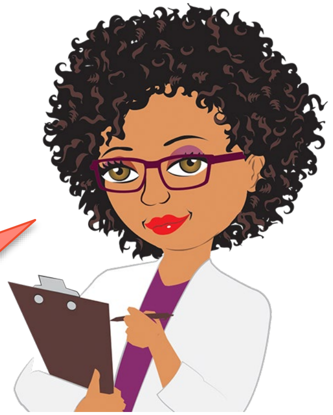

Hmmm, our data are quite complex. How can I be sure that you won't misinterpret the data or conduct misleading analyses?

Case 3: Secondary analyses & protecting the interests of data custodian

I would like to conduct some secondary analyses using your data. Will you share them with me?

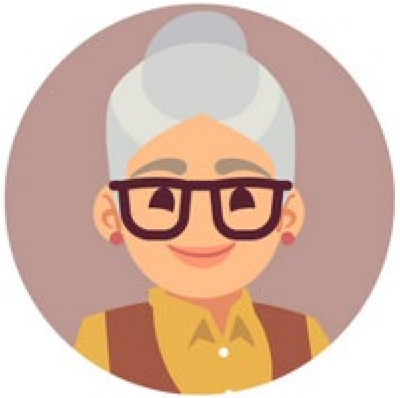

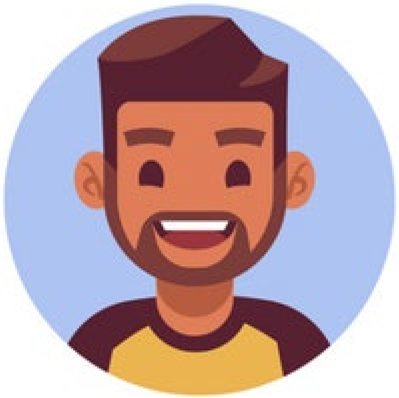

I don’t have the time or resources to prepare the data for you. Do you have any funding to support this? And how will my contributions be recognised?
