# Supplementary material for: Responsibilities for receiving and using individual participant data
Source: Cochrane Evid Synth Methods. 2023 Nov 3;1(9):e12028. doi: 10.1002/cesm.12028 (PMC11795960; doi:10.1002/cesm.12028)
Supplement: Supplementary file 3 — Supporting information. [file CESM-1-e12028-s002.pdf]

APPENDIX 3. Participant demographics (n=16)

	n (%)
Country where based	
Australia	15 (94)
Mexico	1 (6)
Have you ever requested data from another investigator?	
Yes	8 (50)
No	4 (25)
Did not answer	4 (25)
Which of the following best classifies your current primary employer?	
University	11 (69)
Hospital	0 (0)
Commercial sector/industry	0 (0)
Government body	0 (0)
Charities/societies/foundations	0 (0)
Self-employed	0 (0)
Other	1 (6)
Did not answer	4 (25)
What is your career stage?	
Early career researcher (ECR)	6 (38)
Mid career research (MCR)	2 (13)
Senior researcher	2 (13)
Other/not a researcher	2 (13)
Did not answer	4 (25)
Sex	
Male	7 (44)
Female	9 (56)
